# Multiplex Cytokine Analysis of Aqueous Humor in Juvenile Idiopathic Arthritis-Associated Anterior Uveitis With or Without Secondary Glaucoma

**DOI:** 10.3389/fimmu.2018.00708

**Published:** 2018-04-05

**Authors:** Dirk Bauer, Maren Kasper, Karoline Walscheid, Jörg M. Koch, Philipp S. Müther, Bernd Kirchhof, Arnd Heiligenhaus, Carsten Heinz

**Affiliations:** ^1^Department of Ophthalmology and Ophtha-Laboratory at St. Franziskus-Hospital Münster, Münster, Germany; ^2^Center for Ophthalmology, University of Cologne, Cologne, Germany; ^3^Center for Ophthalmology, University of Duisburg-Essen, Essen, Germany

**Keywords:** juvenile idiopathic arthritis-associated anterior uveitis, secondary glaucoma, intraocular pressure, multiplex bead assay analysis, aqueous humor

## Abstract

Patients with juvenile idiopathic arthritis often develop chronic anterior uveitis (JIAU). JIAU patients possess a particularly high risk for developing secondary glaucoma when inflammatory inactivity has been achieved. By using multiplex bead assay analysis, we assessed levels of pro- and anti-inflammatory cytokines, chemokines, or metalloproteinases in the aqueous humor (AH) of patients with clinically inactive JIAU with (JIAUwG) or without secondary glaucoma (JIAUwoG), or from patients with senile cataract as controls. Laser-flare photometry analysis prior to surgery showed no significant differences between JIAUwG or JIAUwoG. Compared with the control group, levels of interleukin-8, matrix metalloproteinase-2, -3, -9, serum amyloid A (SAA), transforming growth factor beta-1, -2, -3 (TGFβ-1, -2, -3), and tumor necrosis factor-alpha in the AH were significantly higher in patients with clinically inactive JIAUwG or JIAUwoG. Samples from JIAwoG patients displayed significantly higher levels of SAA (*P* < 0.0116) than JIAUwG patients. JIAUwG patients showed an increased level of TGFβ-2 in AH samples compared with JIAUwoG (*P* < 0.0009). These molecules may contribute to the clinical development of glaucoma in patients with JIAU.

## Introduction

Approximately 11–13% of patients with juvenile idiopathic arthritis eventually develop uveitis (JIAU), too, typically as chronic anterior uveitis with insidious onset, in the absence of redness or pain. Risk factors for developing uveitis include the presence of anti-nuclear antibodies, oligoarthritis subtype, and early onset of the disease (1). Early onset of uveitis poses a particularly high risk for developing sight-threatening complications, such as synechiae, cataract, glaucoma, and macular edema (2, 3). To prevent such complications, the main goals in treating juvenile idiopathic arthritis (JIA) children include early recognition of uveitis, achieving inflammatory inactivity, preserving vision, and avoiding development of further complications.

Topical treatment with corticosteroids is the first-line therapy, but it may promote elevated intraocular pressure (IOP) and cataract development. Systemic medication with methotrexate, adalimumab, and other disease-modifying anti-rheumatic drugs (DMARDs) can be used if the topical treatment is ineffective or for chronic disease. Uveitis patients have an increased risk for elevated IOP and uveitic glaucoma (UG), which might be induced by the inflammatory disease, but may also result from corticosteroids given as the first treatment approach to reduce ocular inflammation (4). Reported incidence rates of glaucoma in patients with JIAU are up to 38% (5). Mostly, the IOP first increases when inflammatory activity has been eliminated, possibly due to reduced uveoscleral outflow (6). Patients with UG frequently develop very high-IOP values, with peaks of IOP and varying responses to topical anti-glaucomatous drugs, so that about one-third of adult patients and nearly two-thirds of pediatric patients need surgery (7). Many investigators believe that secondary open-angle glaucoma develops as a result of chronic changes to the outflow pathway of the trabecular meshwork (TM). Changes to the TM include deposition of extracellular matrix (ECM), different composition of ECM protein, and loss of trabecular endothelial cells, eventually leading to reduced phagocytosis and altered protein cleavage (4, 8). Another often described, but not clearly confirmed explanation for increasing IOP is mechanical obstruction of the outflow pathway by cells and debris (4, 8, 9). Local immune responses may differ in JIAU patients with (JIAUwG) or without glaucoma (JIAUwoG), thereby mediating different outflow rates of the TM. It has been shown previously that active inflammation may result in increased uveoscleral outflow rates and reduced aqueous humor (AH) production (10).

Such local immune responses may be controlled by proinflammatory and anti-inflammatory cytokines, which can be secreted by immune cells and certain other cell types to regulate inflammation. Proinflammatory cytokines are predominantly produced by helper T cell and macrophages to upregulate inflammatory reactions (11). Therapies against inflammatory diseases often involves monoclonal antibodies targeting proinflammatory cytokines or their receptors. Proinflammatory cytokines [e.g., tumor necrosis factor-alpha (TNF-α) or IL-1β] may activate proforms of metalloproteinases (MMP) which are known to participate in the degradation of ECM and in the regulation of IOP (12). Anti-inflammatory cytokines like transforming growth factor beta (TGF-β) often have both pro-and anti-inflammatory effects. TGF-β may convert an active site of inflammation into one dominated by resolution and repair (13). TGF-β often exhibits disparate effects with immune-enhancing activity in local tissues and immune-suppressive function in the systemic circulation. TGF-β1 and TGF-β2 may increase ECM production in the anterior chamber and also inhibit the degradation of existing ECM, e.g., by downregulating MMPs (14, 15).

We hypothesized that altered outflow rates might be reflected by alterations in the MMP, interleukin (IL)-8, and TGFβ pathways. Therefore, measuring cytokine concentrations in the AH of JIAU patients with and without glaucoma could increase our knowledge of the local pathogenetic processes and may help to explain why some patients with JIAU develop glaucoma and others do not. The multiplex bead immune assay technique allows the simultaneous detection of multiple cytokines even from small clinical samples, as shown previously in patients with uveitis (16–18), branch retinal vein occlusion (19), or primary open-angle glaucoma (POAG) (20). In the present study we used this method to investigate the cytokine profile in patients with clinically inactive JIAU with or without glaucoma.

## Materials and Methods

### Subjects

The study design complies with the standards put forth by the Declaration of Helsinki. The study was approved by the local ethics committee. All subjects provided written informed consent for AH collection during independently planned surgery. For underaged JIAU patients, written informed consent was obtained from the patients’ parents.

Uveitis was classified according to the recommendations put forth by the international uveitis study group (IUSG), under consideration of the recent modifications (21, 22). Laser-flare photometric measurements taken at the final visit before surgery were performed according to previous studies (23–25) using the KOWA FM-500 device (Kowa Company, Electronics and Optics Division, Tokyo, Japan).

Diagnosis and treatment of JIAU followed the German national recommendations for management of disease (26). All patients fulfilled the International League of Associations for Rheumatology (ILAR) criteria for JIA diagnosis (27) and were classified accordingly. Glaucoma diagnosis was established when visual field defects or optic neuropathy were observed. IOP could not be controlled in any of the eyes in the JIAUwG group despite maximally tolerable topical or systemic IOP-lowering therapy or the optic nerve had deteriorated despite apparently normal IOP values. Therefore, standard trabeculectomy to lower IOP was performed in all eyes of the JIAUwG group, and aqueous taps were taken at the beginning. Eyes with JIAUwoG showed no changes at the optic nerve and had not received any topical or systemic IOP-lowering medication at any time during the previous clinical course. In JIAUwoG patients, IOP was never measured above 21 mmHg. Aqueous taps in the JIAUwoG group were taken at the beginning of routine cataract extraction. As a control group, patients undergoing routine surgery for senile cataract were included.

For all groups, suspicion of steroid-induced ocular hypertension constituted an exclusion criterion. None of the JIAU eyes had active inflammation, as defined by anterior chamber (AC) cells ≥0 during the 3 months prior to AH collection, and all had been treated 5× daily with prednisolone acetate 1% for one week prior to surgery. In the control group, any history of systemic or eye inflammation represented an exclusion criterion. Only one eye of an individual patient was included in the study in all groups.

### AH Collection

Aqueous humor samples (100 to 200 µl) were collected at the beginning of the surgery through limbal paracentesis using a 0.5-ml syringe with a 30-gauge needle (BD Hamburg, Germany). Care was taken to prevent contamination with blood or intraocular tissue. All samples were immediately frozen and stored at −80°C until analysis.

### Multiplex Bead Assay

Concentrations of IL-8, monocyte chemoattractant protein-1 (MCP-1), matrix metalloproteinases-1, -2, -3, -9 (MMP-1, -2, -3, -9), serum amyloid A (SAA), TGFβ-1, -2, -3, and TNF-α were measured by multiplex bead assay analysis (Luminex^®^ Performance Assay TGFβ Multiplex Kit R&D Systems Abingdon UK; Procarta^®^ Immunoassay Affymetrix, Santa Clara US) according to the manufacturer’s instructions. Samples were analyzed with LUMINEX^®^ 100/200™ using the Luminex xPonent software (Luminex, USA).

### Statistical Analysis

Statistical analysis was performed using MedCalc statistical software 10.0.1.0 (Marikerke, Belgium). For analysis of normally distributed data, Student’s *t*-test or ANOVA was applied. For categorical values, χ^2^ or Fisher’s Exact test were used. *P* ≤ 0.05 was considered as statistically significant. For multiple comparisons, Bonferroni correction was applied and *P* ≤ 0.0045 was considered as statistically significant. Correlations between different cytokine concentrations, correlation between cytokine concentrations and age, duration of uveitis, and non-invasive laser flare were calculated using Spearmans’s correlations test. Odds ratios (ORs) for the effect of all cyctokines and clinical parameters were calculated using multivariate logistic regression models with backward variable selection.

## Results

### Demographic Data

Overall, 30 JIAU patients, 15 JIAU subjects without glaucoma (JIAUwoG) (average 15.1 years at AH collection) and another 15 JIAU subjects with glaucoma (JIAUwG) (average 13.1 years at AH collection), were included. Additionally, 24 subjects with senile cataract (average 75.5 years at AH collection) were used as controls. The demographic data of the subjects, including number of patients, age, duration of uveitis, lens status, and most recent laser-flare values, are summarized in Table 1.

**Table 1:** Demographic data of patients, treatment, and non-invasive laser-flare measurements.

	C	JIAUwoG	JIAUwG	*P*-value
Number of eyes	24	15	15	
Age (years; mean ± SD)	75.5 ± 9.1	15.1 ± 11.5	13.1 ± 3.4	<0.001*
Duration of uveitis (years, mean ± SD)	n.a.	5.6 ± 4.5	6.9 ± 3.1	0.37^#^
Lens status	n.a.	15 phakic	9 phakic, 3 PC IOL, 3 aphakic	0.023^+^
Posterior synechiae (eyes)	n.a.	13	6	0.03^+^
Systemic corticosteroids	n.a.	6	2	0.21^+^
csDMARD	n.a.	13	13	1^+^
bDMARD	n.a.	3	2	1^+^
Non-invasive laser-flare photometric analysis (KOWA LF-500) (photons/ms, mean ± SD)	n.a.	110.3 ± 40.8	65.5 ± 15.3	0.26^#^

*C, control senile cataract eyes; JIAUwoG, juvenile arthritis-associated anterior uveitis without glaucoma; JIAUwG, juvenile arthritis-associated anterior uveitis with glaucoma; n.a., not applicable; PC, posterior chamber; IOL, intraocular lens; csDMARD, conventional synthetic disease-modifying anti-rheumatic drug; bDMARD, biologic disease-modifying anti-rheumatic drug*.

*ANOVA, ^#^t-test, ^+^χ^2^ test/Fisher’s Exact test.

Age of JIAUwG and JIAUwoG patients did not significantly differ (*P* = 0.5); however, both JIAU groups were significantly younger than the control group with senile cataract (*P* < 0.0001). Furthermore, disease duration of JIAU (time from onset to the date of surgery) in patients without or with glaucoma was not significantly different (*P* = 0.37).

Six JIAUwG patients had received topical prostaglandins (Latanoprost or Bimatoprost) until 2 weeks before glaucoma surgery. Six of 15 JIAUwoG subjects had received steroids systemically, while this was the case in only 2 of 15 JIAUwG patients (Table 1). Of both JIAU groups, 13 of 15 subjects had received conventional synthetic disease-modifying anti-rheumatic drug (csDMARD; methotrexate, cyclosporine A, or azathioprine). Three of 15 subjects with JIAUwoG and 2 of 15 subjects with JIAUwG had received biological disease-modifying anti-rheumatic drug (bDMARD; adalimumab, infliximab, or tocilizumab).

The lens status of the 15 eyes with JIAUwoG were phakic, whereas only 9 of 15 eyes in JIAUwG were phakic, 3 were pseudophakic, and another 3 were aphakic.

Non-invasive laser-flare measurements did not differ between JIAUwoG (110.3 ± 40.8 photons/ms) and JIAUwG (65.5 ± 15.3 photons/ms) patients (*P* = 0.26).

Laser-flare photometric measurement of the six JIAUwG eyes treated with prostaglandins prior to surgery (62.9 ± 19.8 photons/ms) compared with the nine JIAUwoG eyes (68.93 ± 26.2 photons/ms) revealed no differences (*P* = 0.85).

### Differences in the Cytokine Profile in the Anterior Chamber

**Figure 1:**
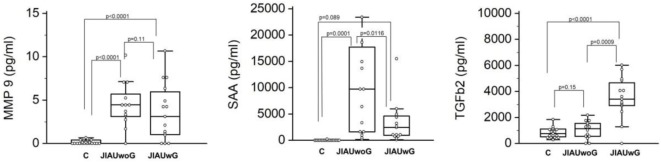
Box plots of matrix metalloproteinases (MMP)-9, serum amyloid A (SAA), and transforming growth factor beta (TGFβ)-2 in patients with juvenile arthritis with (JIAUwG) or without glaucoma (JIAUwoG). Specimens collected from patients with senile cataract served as control. Dots represent individual values. ANOVA with Bonferroni correction (MMP-9, SAA, TGFβ-2: *P* < 0.001). Comparison between two groups by *t*-test.

Compared with the cataract group (control), in whom SAA expression could not be detected, JIAUwoG patients showed an extensive level of SAA (*P* < 0.0001), and JIAUwG displayed an elevated level of SAA when compared with the control group, but this did not reach the level of significance (*P* < 0.089). Furthermore, SAA levels in the AC were significantly higher in JIAUwoG than in JIAUwG patients (*P* < 0.0116; Figure 1).

The control group showed a slight constitutive expression of TGFβ-2. The levels of TGFβ-2 in the JIAUwG patients were significantly higher than in the JIAUwoG patients (*P* < 0.0009) and also compared with the control group (*P* < 0.0001; Figure 1).

**Figure 2:**
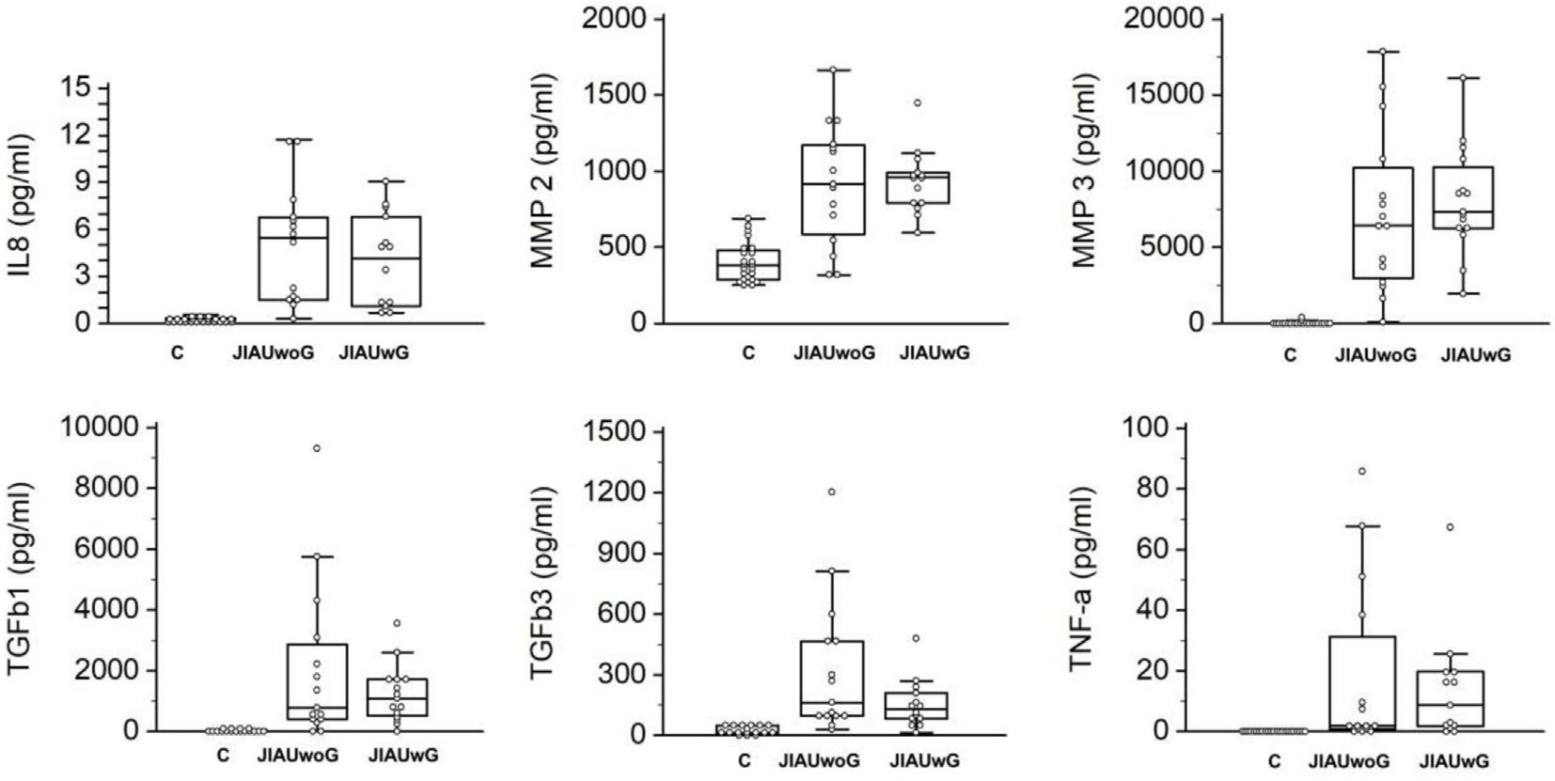
Box plots of interleukin-8 (IL-8), matrix metalloproteinases (MMP)-2, MMP-3, transforming growth factor beta (TGFβ)-1, TGFβ-3, and tumor necrosis factor-alpha (TNF-α) in patients with juvenile idiopathic arthritis-associated uveitis with or without glaucoma. Specimens collected from patients with senile cataract served as a control. Dots represent individual values. ANOVA with Bonferroni correction (IL-8: *P* = 0.004, MMP-2: *P* < 0.001, MMP-3: *P* < 0.001, TGFβ-1: *P* = 0.003, TGFβ-3: *P* = 0.002, TNF-α: *P* = 0.005). Comparison between two groups by *t*-test.

Compared with the cataract group, the concentration of IL-8, MMP-2, MMP-3, MMP-9, TGFβ-1, TGFβ-3, and TNF-α was significantly increased in subjects with JIAUwG or JIAUwoG. However, no significant differences in MCP-1 and MMP-1 were observed between the control group and both JIAU groups (Figure 2).

The correlation among cytokine levels are shown in Table 2. The results show that there is a correlation for the majority of cytokines. However, MCP-1 did not correlate with the other cytokines (Table 2). TGFβ-2 correlated only with TGFβ-1 and with TGFβ-3. A negative correlation was not calculated between any of the various cytokines.

**Table 2:** Correlation between cytokines.

ρ/*P-*value	IL-8	MCP-1	MMP-1	MMP-2	MMP-3	MMP-9	SAA	TGFβ-1	TGFβ-2	TGFβ-3	TNF-α
IL-8		0.199	0.828	0.731	0.827	0.771	0.725	0.489	0.307	0.616	0.695
MCP-1	0.146		0.181	0.187	0.24	0.159	0.086	0.079	0.043	0.042	0.078
MMP-1	<0.0001*	0.1874		0.694	0.802	0.736	0.815	0.536	0.254	0.610	0.679
MMP-2	<0.0001*	0.1728	<0.0001*		0.846	0.574	0.697	0.485	0.33	0.579	0.733
MMP-3	<0.0001*	0.0807	<0.0001*	<0.0001*		0.673	0.781	0.513	0.338	0.59	0.75
MMP-9	<0.0001*	0.2468	<0.0001*	<0.0001*	<0.0001*		0.621	0.499	0.275	0.544	0.656
SAA	<0.0001*	0.53	<0.0001*	<0.0001*	<0.0001*	<0.0001*		0.655	0.298	0.760	0.652
TGFβ-1	0.009*	0.5923	0.003*	0.001*	0.0005*	0.0007*	<0.0001*		0.79	0.846	0.351
TGFβ-2	0.376	0.7714	0.085	0.0251	0.0218	0.0624	0.0433	<0.0001*		0.593	0.261
TGFβ-3	<0.0001*	0.7781	<0.0001*	0.0001*	0.0001*	0.0002*	<0.0001*	<0.0001*	0.0001*		0.423
TNF-α	<0.0001*	0.57	<0.0001*	<0.0001*	<0.0001*	<0.0001*	<0.0001*	0.0172	0.0763	0.0042	

*Significant positive correlations were calculated for the majority of cytokines. Monocyte chemoattractant protein-1 (MCP-1) and transforming growth factor beta-2 (TGFβ-2) concentrations did not significantly correlate with the other cytokines. Correlation coefficient (ρ) and *P*-values for each pair of cytokines were calculated by using Spearman’s correlation test*.

*Significant when P < 0.0045 after Bonferroni correction for multiple comparisons.

*IL-8, interleukin-8; MCP-1, monocyte chemoattractant protein-1; SAA, serum amyloid A; TGFβ-1, transforming growth factor beta-1; TNF-α, tumor necrosis factor-alpha*.

### Correlation of Cytokines With Age, Duration of Uveitis, and Laser-Flare Values

Correlation of cytokines in the two JIAU groups showed no significant differences with regard to age, uveitis duration, or laser-flare values. Only MMP-2 was significantly elevated in patients with increased laser-flare photometric values (*P* = 0.0038; Table 3).

**Table 3:** Correlation of cytokines with age, duration of uveitis, or laser-flare values.

	Age (years)		Duration of uveitis (years)		Laser-flare photometric values (photons/ms)	
	Correlation coefficient	*P*-value	Correlation coefficient	*P*-value	Correlation coefficient	*P*-value
IL-8	−0.21	0.25	−0.078	0.69	0.171	0.41
MCP-1	−0.364	0.05	−0.167	0.36	0.35	0.09
MMP-1	0.033	0.85	−0.042	0.81	0.256	0.22
MMP-2	−0.227	0.221	−0.3	0.16	0.603	0.0038*
MMP-3	0.006	0.97	−0.021	0.90	0.512	0.014
MMP-9	−0.298	0.10	−0.131	0.48	0.227	0.27
SAA	−0.063	0.73	−0,254	0.17	0.399	0.056
TGFβ-1	0.128	0.48	0.107	0.56	0.0038	0.98
TGFβ-2	0.147	0.43	0.185	0.31	0.039	0.85
TGFβ-3	0.161	0.93	−0.122	0.51	0.115	0.58
TNF-α	−0.181	0.32	−0.239	0.19	0.36	0.084

*The correlation coefficient and P-values for cytokines and age, duration of uveitis, and laser-flare values were calculated by Spearman’s correlation test for JIAU eyes. The tests point to a correlation of MMP-2 with the laser-flare values*.

*P < 0.0045 after Bonferroni correction.

*IL-8, interleukin-8; MCP-1, monocyte chemoattractant protein-1; SAA, serum amyloid A; TGFβ-1, transforming growth factor beta-1; TNF-α, tumor necrosis factor-alpha*.

### Impact of Anti-Inflammatory or Anti-Glaucoma Medication on the Expression of Cytokines in the Anterior Chamber

No difference in cytokine levels were observed in JIAU eyes whether patients were treated with csDMARD or prostaglandins. However, in patients treated with bDMARD, we detected significantly higher levels of MMP-1 (*P* = 0.027) and TGFβ-1 (*P* = 0.0059; Table 4).

**Table 4:** Influence of csDMARD, bDMARD, or prostaglandin medication on the expression of cytokines in the anterior chamber.

	csDMARD			bDMARD			Prostaglandins		
	Neg	Pos	*P*-value	Neg	Pos	*P*-value	Neg	Pos	*P*-value
Number	4	26		25	5		9	6	
IL-8	4.2 ± 3.2	7.1 ± 9.5	0.56	7.3 ± 9.6	3.3 ± 2.7	0.37	7.4 ± 12.7	4.7 ± 2.3	0.61
MCP-1	110.1 ± 35.5	160 ± 100	0.33	158 ± 97	130 ± 91	0.56	141 ± 72	151 ± 65	0.79
MMP-1	11.2 ± 8.5	196 ± 807	0.65	38.4 ± 58.9	838.45	0.027	12.7 ± 6.3	30.7 ± 41.3	0.21
MMP-2	821 ± 363	984 ± 380	0.42	1,014 ± 376	704 ± 280	0.09	1,099 ± 428	875 ± 192	0.2567
MMP-3	6,211 ± 4,074	7,924 ± 4,526	0.48	8,210 ± 4,525	5,124 ± 3,200	0.16	7,779 ± 3,522	8,574 ± 3,794	0.68
MMP-9	2.3 ± 2.1	5.5 ± 5.3	0.25	5.5 ± 5.3	2.8 ± 3	0.28	4.1 ± 3.9	2.9 ± 1.9	0.54
SAA	2,074 ± 2,034	7,166 ± 7,433	0.18	6,648 ± 7,002	5,686 ± 8,705	0.78	3,708 ± 4,723	2,663 ± 2,240	0.62
TGFβ-1	311 ± 250	1,844 ± 2,053	0.15	1,213 ± 1,261	3,775 ± 3,471	0.0059	1,026 ± 8,48	1,511 ± 1,087	0.35
TGFβ-2	1,188 ± 1,243	2,643 ± 1,877	0.14	2,448 ± 1,987	2,456 ± 1,147	0.99	3,047 ± 1,751	4,180 ± 1,074	0.18
TGFβ-3	76 ± 32	289 ± 291	0.16	252 ± 299	301 ± 171	0.73	195 ± 244	201 ± 154	0.96
TNF-α	19 ± 32	22 ± 32	0.87	25 ± 33	5.3 ± 3.2	0.2	28 ± 41	21 ± 24	0.71

*Mean values for cytokine concentrations of eyes are depicted without or with systemic or topical treatment, csDMARD, and bDMARD for all eyes with JIAUwG and prostaglandins. Differences in cytokine expression with or without medications were calculated by Student’s t-test*.

*IL-8, interleukin-8; MCP-1, monocyte chemoattractant protein-1; SSA, serum amyloid A; TGFβ-1, transforming growth factor beta-1; TNF-α, tumor necrosis factor-alpha*.

### Risk Factors for the Development of Secondary Glaucoma

Statistical calculation for clinical factors associated with a higher risk for glaucoma manifestation by using univariate regression analysis of age of patients, duration of uveitis, systemic treatment with conventional immunosuppressant (methotrexate, azathioprine, or cyclosporine A) or treatment with adalimumab revealed no significant influence. The presence of posterior synechiae at the time of surgery was associated with a decreased risk for secondary glaucoma development (OR 0.1025, 95% CI 0.0168–0.6280; *P* = 0.013). The multivariate analysis of the above-mentioned factors also showed significant decreased OR for posterior synechiae (OR 0.1026, 95% CI 0.0168–0.628; *P* = 0.0138). Multivariate regression analysis for UG development including all cytokines revealed a significant correlation of TGFβ-2 with UG (OR 1.0009, 95% CI 1.0002–1.0015; *P* = 0.0075). Additional analysis of the other clinical parameters in the multivariate analysis revealed no significance.

## Discussion

The present study investigated the expression of pro- and anti-inflammatory cytokines, chemokines, or metalloproteinases in the AH of patients with clinically inactive uveitis associated with JIA, both with or without glaucoma. Quiescence of uveitis disease was confirmed by clinical observation and ensured by treatment with individually adjusted anti-inflammatory medication before surgery.

We found that the concentrations of IL-8, MMP-2, MMP-3, MMP-9, TGFβ-1, TGFβ-2, TGFβ-3, SAA, and TNF-α were significantly elevated in JIAU samples compared with the control group (senile cataract patients). These results show that even patients with clinically inactive JIAU receiving intensive anti-inflammatory treatment may not achieve an entirely inactive state from an immunological point of view. Instead, the AH in JIAU eyes displayed a constitutive expression of the proinflammatory cytokines IL-8 and TNF-α. We also found an increased expression of MMP-2, MMP-3, MMP-9, TGFβ-1, and TGFβ-3 in eyes with JIAU, independent of the presence of glaucoma. Eyes with JIAUwG displayed increased levels of TGFβ-2, while eyes with JIAUwoG showed significantly increased levels of SAA.

Elevated levels of TGFβ-2 have previously been strongly correlated with increased outflow resistance and IOP elevation in POAG. TGFβ-2 is an anti-proliferative and anti-inflammatory cytokine, which has been discussed in the context of various disorders, including glaucoma (28–30). These findings were supported by *ex vivo* studies using cultured AC systems derived from humans, monkeys, or pigs (31–33). TGFβ-2 was shown to markedly elevate IOP, possibly *via* a mechanism involving increased expression and deposition of extracellular material within the TM (31). An increased TGFβ-2 protein content in the AH *in vivo* also correlated with elevated IOP in rodents (34–37). TGFβ-2 induces increased ECM synthesis in TM cells that is largely regulated by Smad3-mediated signal mechanisms (35, 38, 39).

Increased SAA concentrations in the AC of eyes with glaucoma have been found previously, too. Here, the expression of SAA2 was increased in TM tissues from donors with glaucoma. Furthermore, treatment of cultured human TM cells with recombinant SAA affected upregulation of IL-8 and increased IOP in the human ocular perfusion organ culture (40).

In a recent study, various inflammatory cytokines in the AH of patients with POAG, exfoliation glaucoma (EXG) and senile cataract were determined. Herein, increased TGFβ-1, IL-8, and SAA levels were found in POAG and EXG when compared with the cataract control group. IL-6 was decreased in the POAG group. The levels of TGFβ-1, IL-8, and SAA correlated positively with each other, with IOP, and the number of glaucoma medications. The authors concluded that TGFβ-1, IL-8, and SAA have critical roles in IOP elevations in patients with POAG (20). Another group found the levels of IL-8, TGFβ-1, TNF-α, and SAA to be significantly increased in POAG and pseudoexfoliation glaucoma (PEXG) patients compared with controls, while levels of IL-6 were significantly decreased in both glaucoma groups compared with controls (41).

Moreover, previous studies of rheumatoid arthritis have shown that SAA in the serum was strongly associated with disease activity, but also with the risk of cardiovascular and renal involvement and could, therefore, serve as a marker to help identify patients with persistent inflammation and at risk of extra-articular complications (42). Similar results were found in patients with ankylosing spondylitis (43). C-reactive protein (CRP) and SAA were shown to be valuable biomarkers of infectious or inflammatory processes (44).

It has been shown previously that SAA may attract migrating phagocytes to the site of inflammation, serve as an agonist for toll-like receptor 4, activate nuclear factor NF-κB, and induce expression of both inflammatory cytokines and M2 macrophage markers and to influence polymorphonuclear neutrophils (PMN) by inhibiting apoptosis and enhancing efferocytosis (45, 46). We conclude based on the known activities that SAA is expressed locally in chronic inflammatory conditions such as JIAU and may act via diverse proinflammatory functional mechanisms to regulate homeostasis in the AC of eyes with JIAU and does not support development of glaucoma.

However, inflammation responses in the eye must be downregulated, for example, by anti-inflammatory cytokines, to minimize tissue damage in the eye. It has been shown previously that TGFβ-2 may influence the levels of SAA. A previous study has shown that IL-10−/− mice that develop a form of inflammatory bowel disease (a model for Crohn’s disease) gained more weight and developed lower pathological scores, lower TNF-α concentrations, and lower SAA content when mice were fed a TGFβ-2-containing diet (47). We, therefore, assume that there is a reciprocal relationship between SAA and TGFβ-2 in the AH of JIAU patients. In some of the JIAU patients, high TGFβ-2 levels could be produced to control SAA, but this process might promote the development of glaucoma.

Some of the patients with JIAUwG received medication with Latanoprost or Bimatoprost to treat elevated IOP. Previous studies have shown that topical treatment with Latanoprost decreased levels of TGFβ-1, MMP-2, and tissue inhibitor of metalloproteinases-2 in patients suffering from PEXG (48). In a TM *in vitro* system, Latanoprost decreased collagen accumulation but promoted a contractile phenotype in TM cells that could modulate the conventional outflow pathway (49). Another study has shown that long-term treatment with Latanoprost could be associated with enhanced Tenon’s capsule fibroblast proliferation and increased expression of TGFβ and MMPs, probably promoting the scarring of the filtering blebs following glaucoma-filtering surgery (50). An ELISA assay in patients with glaucoma and prostaglandin therapy revealed no differences in MMP-2 and -9 levels compared with control eyes (51), nor did we find an influence of prostaglandin medication on the expression of cytokines, chemokines, or MMPs in the AC. Therefore, we assume that the modulated cytokine levels in the AH are primarily related to the increased IOP values or development of glaucoma rather than to the prostaglandin/Latanoprost medication.

No information has been published about the impact of csDMARD on intraocular cytokine concentrations. However, our data show that treatment with csDMARD did not significantly alter cytokine concentrations in the AH. In contrast, our data demonstrate that there was a significant difference in the expression of MMP-1 or TGFβ-1 after treatment with bDMARD. An explanation could be that bDMARD are favored in patients with a particularly severe JIAU disease course with or without glaucoma. However, our results imply that the use of bDMARD modulated the local immune response of the patients.

Tumor necrosis factor-alpha is a proinflammatory cytokine with pleiotrophic effects on various cell types. Recent results suggest that the TNF-α neutralizing function dictates not only the success of anti-inflammatory treatment, but also other biological effects. Anti-TNF-α medications are increasingly being used to treat JIAU (52). However, the TNF-targeting molecules available differ in their function to induce complement-dependent cytotoxicity or antibody-dependent cellular cytotoxicity and to trigger full transmembrane TNF-α signaling, which may lead to TGFβ synthesis. It has been described previously that reverse signaling of transmembrane TNF-α may lead to TGFβ production in macrophages, and anti-TNF-α agents selectively trigger this pathway (53).

In some diseases, e.g., rheumatoid arthritis, TGFβ signaling is enhanced and seems to play a proinflammatory rather than an anti-inflammatory role. TGFβ-1 promoted the expression of proinflammatory cytokines and metalloproteinases (54), by acting as a potent chemoattractant for PMN (55), by activating the expression of chemokines (56), and by promoting angiogenesis and synovial cell proliferation (53, 57).

Recent studies have shown that pharmacological blockade of IL-6 by a humanized anti-IL-6 receptor antibody is beneficial in patients with rheumatoid arthritis and JIA, suggesting that IL-6 might be an essential key cytokine in these diseases. IL-6 was shown to be involved in the induction of Th17 cell stimulation with TGFβ. Together with TNF-α and IL-1, IL-6 induces CRP or SAA signaling. Indeed, IL-6 was among those cytokines previously detected in the AH of JIAU patients (58), and while blocking TNF-α reduced acute phase proteins (CRP, SAA), IL-6 blockade additionally induced normalization of these proteins (53, 59).

In conclusion, the results of the current study show that pro- and anti-inflammatory cytokine, chemokine, or metalloproteinase levels are increased in clinically inactive JIAU eyes, suggesting that in these eyes disease is seemingly not inactive from an immunological point of view and that the eyes show a cytokine profile typical of chronic inflammation. The pathogenetic process for the development of glaucoma in some of the JIAU patients may be related to the severity of ocular inflammation, the use of corticosteroids, and complications such as posterior synechiae, damage of anterior chamber angle or to the TM. We therefore conclude that the etiologic mechanisms involved are multifactorial. However, the significantly increased levels of SAA in JIAUwoG and of TGFβ-2 in JIAUwG suggest that the cytokines could play important roles in modulating IOP. Previous studies have found that SAA may induce IL-8 production of TM. TGFβ-2 has been shown to decrease SAA and TNF-α and to regulate ECM synthesis and remodeling (40, 47). These molecules may be relevant for studying the pathologic mechanism of IOP elevation in secondary glaucoma in the future.

## Ethics Statement

The study design complies with the standards put forth by the Declaration of Helsinki. The study was approved by the local ethics committee. All subjects provided written informed consent for AH collection during independently planned surgery. For underaged JIAU patients, written informed consent was obtained from the patients’ parents.

## Author Contributions

DB, MK, and CH designed the study and wrote the manuscript. DB, MK, PM, BK, KW, and CH performed the experiments and analyzed the data. KW, JK, AH, and CH performed the eye surgery, collected the aqueous humor, and provided clinical data. All authors approved the final version of the manuscript.

## Conflict of Interest Statement

The authors declare that the research was conducted in the absence of any commercial or financial relationship that could be construed as a potential conflict of interest.
